# Estimation of Potential Availability of Essential Oil in Some Brands of Herbal Teas and Herbal Dietary Supplements

**DOI:** 10.1371/journal.pone.0130714

**Published:** 2015-06-25

**Authors:** Radosław Kowalski, Tomasz Baj, Grażyna Kowalska, Urszula Pankiewicz

**Affiliations:** 1 Department of Analysis and Evaluation of Food Quality, University of Life Sciences in Lublin, Lublin, Poland; 2 Institute of Agrophysics, Polish Academy of Sciences, Lublin, Poland; 3 Department of Pharmacognosy with Medicinal Plant Unit, Medical University in Lublin, Lublin, Poland; 4 Central Laboratory of Agroecology, University of Life Sciences in Lublin, Lublin, Poland; Agricultural University of Athens, GREECE

## Abstract

**Introduction:**

The aim of the study was to estimate potential availability of essential oil in some brands of herbal products.

**Methods:**

A comparison was performed on the basis of the essential oil yield in the unprocessed raw materials such as leaves of peppermint and lemon balm and inflorescence of chamomile as well as herbal tea bags and in dietary supplements. The yield of essential oil was determined by distillation. Essential oil was analyzed by GC-FID and GC-MS.

**Results:**

It was found that the average potential availability of essential oils in the products such as dietary supplements for the doses recommended by the producers is lower than in the corresponding tea infusions: for peppermint formulations approximately 6-fold lower, for the formulations with lemon balm about 4-fold lower, and for the chamomile preparations about 3-fold lower. It was found that essential oils extracted from herbal teas have a similar chemical profile with characteristic deviations in the amount of individual components, which arise from the origin of the raw material.

**Discussion:**

In contrast to homogenous pharmaceutical herbal mixtures consistent with, the Pharmacopoeia requirements, herbal teas (available in grocery stores) and dietary supplements are often out of control in terms of the yield and composition of the essential oil, which is primarily responsible for the health benefits and aromatic qualities of these products. Analysis of the composition of the dietary supplements showed that they contain on average significantly lower amounts of plant material compared to the herbal teas.

## Introduction

The yield of essential oils is one of the fundamental factors affecting the quality of herb teas or dietary supplements containing such raw materials. Many essential oils have medicinal properties which make them useful in the treatment of various diseases and pathological conditions [1]. The oils are characterized by a broad spectrum of activity, i.e. antibacterial, anti-fungal, anti-parasitic, anti-inflammatory, diuretic, cholagogic, apoflegmatic, irritating, lowering the activity of the central nervous system, soporific, sedative or stimulating, anti-depressive, anticonvulsive, and also as an antioxidant (scavengers of free peroxide radicals), immuno-stimulating and anti-carcinogenic [2, 3].

In view of the above, the objective of the undertaken study was to estimate potential availability of essential oil in some brands of herbal products. A comparison was performed on the basis of the yield of essential oil in the unprocessed raw materials, such as the leaf of peppermint, leaf of lemon balm and inflorescence of chamomile in relation to the herbal tea bags used for brewing and in dietary supplements whose composition included those herbal raw materials.

## Materials and Methods

### Ethics Statement

The study was carried out on private land. The owner of the land gave permission to conduct the study on this site:

1. no specific permissions were required for these locations/activities,
2. the field studies did not involve endangered or protected species.

### Plant Materials and Products

The experimental material consisted of the following raw materials and products:

- Leaves of peppermint (*Mentha piperita* L.), leaves of lemon balm (*Melissa officinalis* L.) and inflorescence of chamomile (*Matricaria chamomilla* L.), acquired from retail shops in the Lublin Region (eastern Poland) and from cultivation in an allotment garden (Lubartów, N 51°27' 41.0394", E 22°37' 5.8152", located in the eastern part of the Lubartów Upland which forms a part of the much larger South Podlasie Lowland). Leaves and inflorescences were harvested at maturity pooled (June 2013). Lemon balm and mint acquired in the second year of cultivation, while chamomile are obtained from annual plants. The plant material is dried in a room free from air circulation in low temperature.
- Herbal tea bags: peppermint, lemon balm, and chamomile, purchased in Lublin markets.
- Dietary supplements (in the form of capsules, liquids and powders), containing peppermint, lemon balm and chamomile in their composition, purchased in Lublin pharmacies.

A listing of the raw materials and products is given in Table 1.

**Table 1:** Unprocessed materials and products used in the study.

Component	Group	Form processing raw material/product	The content of the ingredient in the minimum dose (serving)	Code
***Mentha piperita***	unprocessed material	dry material	not applicable	cMT1
			not applicable	cMT2
			not applicable	cMT3
			not applicable	cMT4
	herbal tea	tea bags to brew	2000 mg	tMT1
			2000 mg	tMT2
			2000 mg	tMT3
			2000 mg	tMT4
			2250 mg	tMT5
			2000 mg	tMT6
			2000 mg	tMT7
			2000 mg	tMT8
	dietary supplement	tablets	1000 mg	sMT1
		capsules	820 mg	sMT2
		capsules	28,3 mg	sMT3
		tablets	1000 mg	sMT4
		liquid	130 mg	sMT5
		tablets	9 mg	sMT6
		tablets	60 mg	sMT7
		tablets	150 mg	sMT8
		tablets	1000 mg	sMT9
		tablets	350 mg	sMT10
***Melissa officinalis***	unprocessed material	dry material	not applicable	cML1
			not applicable	cML2
			not applicable	cML3
			not applicable	cML4
	herbal tea	tea bags to brew	1500 mg	tML1
			2000 mg	tML2
			2000 mg	tML3
			2000 mg	tML4
			1700 mg	tML5
			2000 mg	tML6
			2000 mg	tML7
			2000 mg	tML8
	dietary supplement	tablets	50 mg	sML1
		capsules	600 mg	sML2
		liquid	150 mg	sML3
		liquid	750 mg	sML4
		powder	1380 mg	sML5
		tablets	1050 mg	sML6
		tablets	50 mg	sML7
		capsules	30 mg	sML8
		powder	1250 mg	sML9
		capsules	3000 mg	sML10
***Matricaria chamomilla***	unprocessed material	dry material	not applicable	cMC1
			not applicable	cMC2
			not applicable	cMC3
			not applicable	cMC4
	herbal tea	tea bags to brew	1500 mg	tMC1
			1500 mg	tMC2
			1500 mg	tMC3
			1500 mg	tMC4
			2000 mg	tMC5
			1750 mg	tMC6
			1500 mg	tMC7
			1750 mg	tMC8
	dietary supplement	liquid	200 mg	sMC1
		capsules	147 mg	sMC2
		capsules	140 mg	sMC3
		liquid	375 mg	sMC4
		capsules	200 mg	sMC5
		tablets	150 mg	sMC6
		capsules	675 mg	sMC7
		liquid	1000 mg	sMC8
		powder	1500 mg	sMC9
		capsules	1300 mg	sMC10

### Qualitative and Quantitative Analysis of Essential Oil

#### Assay of essential oil yield

The yield of essential oil in the raw materials and in the herbal teas was analysed with the method of distillation. With the food-product character of herbal teas and oral application of dietary supplements it can be assumed that the essential oil contained in them is absorbed by the human organism and constitutes a quantitatively potentially available level of biologically active substances that may exhibit activity within the broad spectrum of their properties. The potential availability of essential oil after consuming such teas and dietary supplements was determined with the method of calculation on the basis of the minimum and maximum dose (portion) recommended by the producers, with the assumption that the yield of essential oil in the herbal component corresponds to the average yield of that component in the respective herbal tea.

The distillation of peppermint and chamomile essential oils (raw materials and herbal teas) was conducted in a Clevenger-type apparatus, according to the Polish Pharmacopoeia VIII [4]. The time of the distillation was deemed appropriate for the procedure contained in the detailed monograph and amounted to 3 hours for peppermint and 2 hours for chamomile.

For lemon balm (raw materials and herbal teas), due to the lack of a detailed monograph in PP VIII [4], we referred to the monograph in PP VI [5], where the distillation time is 3 hours. The method of the direct distillation was applied for lemon balm, and the indirect method (with xylene) was used for chamomile and peppermint. For this purpose, 20.0 g of inflorescences or leaves, 400 ml water and 0.30 ml of xylene was used. A flask of 1000 cm^3^ was used. Water was poured onto the weighed material in the flask and then the xylene was added to the flask. After being brought to the boil and the first drop being distilled the flask continued to be heated for 3 h. 30 min after finishing distillation the result was read and xylene correction (0.26 ml) was subtracted from the reading. The measured essential oil volume was counted for 100 g of material.

The distillation was carried out in triplicate. The oil was recovered directly and was stored with anhydrous sodium sulfate in dark vials at 4°C.

#### GC analysis

Essential oil (10 μl) was diluted 1:100 using *n*-hexane to achieve 1 ml volume, then 100 μl of C12, and C19 as internal standards mixture solution (1 mg/ml in toluene) was added to the diluted oil. Such prepared samples were subjected to GC-MS and GC-FID evaluations. Chromatographic separations were performed in triplicate.

Reproducibility has been evaluated for the chromatographic analysis of volatile compounds standards in order to verify the accuracy of the chromatographic separation. Carried out after 10 measurements. Then, in order to detect and eliminate questionable results, the data obtained was verified using the Grubbs test. The values of the coefficients of variation for the reproducibility of the method ranged from 0.00% to 9.10%, so they were less than 10%, which demonstrates the reproducibility of the method and its usefulness in the experiment carried out [6].

#### GC/MS

GC-MS: ITMS Varian 4000 GC-MS/MS (Varian, USA) equipped with a CP-8410 auto-injector and a 30 m x 0.25 mm VF-5ms column (Varian, USA), film thickness 0.25 μm, carrier gas He 0.5 ml/min, injector and detector temperature were, respectively, at 250 and 200°C; split ratio 1:50; inject volume 5 μl. A temperature gradient was applied (50°C for 1 minute, then increased by 4°C/min to 250°C, 250°C for 10 minutes); ionization energy 70 eV; mass range: 40–870 Da; scan time 0.80 s. Data acquisition and processing, and instrumental control were performed by the Varian MS Workstation Version 6.42.

#### GC/FID

GC Varian 3800 (Varian, USA) equipped with a CP-8410 auto-injector and a 30 m x 0.25 mm DB-5 column (J&W Scientific, USA), film thickness 0.25 μm, carrier gas He 0.5 ml/min, injector and detector FID temperatures were, 260°C; split ratio 1:100; inject volume 5 μl. A temperature gradient was applied (50°C for 1 minute, then increased by 4°C/min to 250°C, 250°C for 10 minutes).

#### Qualitative analysis

The qualitative analysis was carried out on the basis of MS spectra which were compared with the spectra library by means of the NIST MS Search Program (NIST 08, Software Version 2.0f) [7], and with the data available in literature [8, 9]. Identity of the compounds was confirmed by their retention indices [10], taken from literature [8, 9], and our own data for standards (1,8-cineole, chamazulene, eugenol, limonene, menthone, thymol, carvone, carvacrol, menthol, caryophyllene oxide, E-caryophyllene from Fluka, Sigma-Aldrich Chemie GmbH, Germany).

#### Quantitative analysis

The percentage of main components of the essential oil was presented assuming that the sum of peak areas for all identified constituents is 100%.

Only main components of essential oil were selected on a base of the concentration criterion–>1.0%, although in the one essential oil.

#### Statistical Methods

All variables showed normal distribution, when analyzed using the Shapiro-Wilk normality test, and therefore, the parametric statistical analysis was used. Data were expressed as mean ± standard error of mean. The variables were compared among groups by one-way analysis of variance (ANOVA). For cases in which some significance was shown, post hoc Duncan’s test was used. For all analyses, we adopted the significance level of *P* < 0.05. The software used for statistical analysis was SAS statistical system (SAS Version 9.1, SAS Inst., Cary, N.C., U.S.A.).

## Results and Discussion

Table 2 presents the yield of essential oil in non-fragmented unprocessed raw materials such as the leaves of peppermint, leaves of lemon balm, and inflorescence of chamomile, and in herbal teas from those herb species. Table 3 presents data concerning the potential availability of essential oil after the consumption of the herbal teas and dietary supplements.

**Table 2:** The yield of essential oils in the analyzed unprocessed materials and tea bags.

Yield of essential oil [% v/w±SD]					
*Mentha piperita* (*F* = 1130,94**)		*Melissa officinalis* (*F* = 84.38***)		*Matricaria chamomilla*(*F* = 71,38***)	
cMT1^#^	1.32 ± 0.04e	cML1	0.18 ± 0.01ab	cMC1	0.43 ± 0.00f
cMT2	1.84 ± 0.04c	cML2	0.17 ± 0.01bc	cMC2	0.41 ± 0.04f
cMT3	2.19 ± 0.05b	cML3	0.19 ± 0.01a	cMC3	0.74 ± 0.04a
cMT4	2.48 ± 0.05a	cML4	0.18 ± 0.01ab	cMC4	0.45 ± 0.04ef
average cMT	1.96	average cML	0.18	average cMC	0.51
PPVIII norm	>1.20	PPVI norm	>0.05	PPVIII norm	>0.40
tMT1	1.61 ± 0.03d	tML1	0.12 ± 0.02e	tMC1	0.56 ± 0.03cb
tMT2	1.31 ± 0.04e	tML2	0.08 ± 0.01f	tMC2	0.48 ± 0.03de
tMT3	0.87 ± 0.03g	tML3	0.16 ± 0.02cd	tMC3	0.49 ± 0.01de
tMT4	1.28 ± 0.03e	tML4	0.09 ± 0.01f	tMC4	0.50 ± 0.01ef
tMT5	0.25 ± 0.00j	tML5	0.09 ± 0.01f	tMC5	0.35 ± 0.00g
tMT6	1.11 ± 0.03f	tML6	0.14 ± 0.01d	tMC6	0.33 ± 0.03g
tMT7	0.58 ± 0.03h	tML7	0.07 ± 0.01f	tMC7	0.54 ± 0.00c
tMT8	0.52 ± 0.03i	tML8	0.17 ± 0.01ab	tMC8	0.59 ± 0.01b
average tMT	0.94	average tML	0.12	average tMC	0.48
cMT/tMT	2.09	cML/tML	1.50	cMC/tMC	1.06
PPVIII norm	>0.90	PPVI norm	>0.05	PPVII norm	>0.40

The *F* values (one way ANOVA) are shown in the table. The levels of significance are indicated by an asterisk (**0.001 <*P*≤0,01; ***P ≤0,001).

^#^Designation according to Table 1.

a, b, c …—values designated with the same letters (a, b, c, d) within columns do not significantly differ at 5% error (Duncan’s test).

**Table 3:** The potential availability of essential oil for consumption of herbal teas and dietary supplements.

The potential availability of essential oil [mg]								
*Mentha piperita*			*Melissa officinalis*			*Matricaria chamomilla*		
Product	Min (*F* = 1645,4**)	Max (*F* = 1759,7**)	Product	Min (*F* = 111,3**)	Max (*F* = 113,9**)	Product	Min (*F* = 651,2**)	Max (*F* = 713,1**)
tMT1^#^	32.20a	96.60a	tML1	1.80d	5.40c	tMC1	8.40b	25.20b
tMT2	26.20b	78.60b	tML2	1.60def	4.80cd	tMC2	7.20cd	21.60cd
tMT3	17.40d	52.20d	tML3	3.20b	9.60a	tMC3	7.35cd	22.05cd
tMT4	25.60b	76.80b	tML4	1.80d	5.40c	tMC4	7.50c	22.50c
tMT5	5.62i	16.88h	tML5	1.53def	4.59cde	tMC5	7.00d	21.00d
tMT6	22.20c	66.60c	tML6	2.80c	8.40b	tMC6	5.78f	17.32e
tMT7	11.60e	34.80e	tML7	1.40ef	4.20def	tMC7	8.10b	24.30b
tMT8	10.40f	31.20f	tML8	3.40ab	10.20a	tMC8	10.32a	30.98a
average tMT	18.90	56.7	average tML	2.19	6.57	average tMC	7.71	23.12
sMT1	9.40g	18.80h	sML1	0.06h	0.22i	sMC1	0.96j	2.88i
sMT2	7.71h	23.12g	sML2	0.72g	0.72i	sMC2	0.71j	2.11ij
sMT3	0.27m	1.60k	sML3	0.18h	0.36i	sMC3	0.67j	6.05g
sMT4	9.40g	18.80h	sML4	0.90g	0.27g	sMC4	1.80i	5.40gh
sMT5	1.22kl	2.44k	sML5	1.66de	1.66h	sMC5	0.96j	0.96k
sMT6	0.08m	1.03k	sML6	1.26f	3.78f	sMC6	0.72j	1.44jk
sMT7	0.56lm	5.08j	sML7	0.06h	0.36i	sMC7	3.24h	12.96f
sMT8	1.41k	2.82k	sML8	0.04h	0.04i	sMC8	4.80g	4.80h
sMT9	9.40g	18.80h	sML9	1.50def	4.50cdef	sMC9	7.20cd	21.60cd
sMT10	3.29j	9.87i	sML10	3.60a	3.60f	sMC10	6.24e	12.48f
average sMT	4.27	10.24	average sML	1.00	1789	average sMC	2.73	7.07
tMT/sMT	4.42	5.54	tML/sML	2.19	3.67	tMC/sMC	2.82	3.27

The *F* values (one way ANOVA) are shown in the table. The levels of significance are indicated by an asterisk

**0.001 <*P*≤0,01;

***P ≤0,001.

^#^Designation according to Table 1.

a, b, c …—values designated with the same letters (a, b, c, d) within columns do not significantly differ at 5% error (Duncan’s test).

### Peppermint

The yield of essential oil in the unprocessed raw material—leaf of peppermint, varied from 1.32% to 2.48% (mean of 1.96%). Leaves of peppermint can contain from 0.5 to 5.0% of oil [11]. The pharmacopeial raw material of peppermint *Mentha piperita* L. consists of whole or shredded dried leaves which should contain no less than 1.2% of the essential oil for non-fragmented plant material and a lot less than 0.9% in the case of shredded plant material [4]. The criterion of Polish Pharmacopoeia VIII [4] concerning the yield of volatile substances was met by five herbal teas. The concentration of essential oil in the herbal teas analysed varied from 0.25 to 1.61% (mean of 0.94%) and on average it was circa twice lower than in the unprocessed material (Table 2). The difference between the extreme concentrations was 2.23%, which may largely be an effect of raw material processing during production of the herbal teas, aside from its possibly quite varied initial quality.

The yield of volatile substances is affected by the degree of fragmentation of the raw material. The process of fragmentation results in the destruction of secretory structures and liberation of essential oil in the course of the technological process, and thus reduces the yield of essential oil in the final product. As Kowalski and Wawrzykowski report [12], the process of granulation, to which herbal materials can be subjected for the production of herbal teas for the purpose of homogenisation of a batch of raw material, may lead to a notable reduction of the yield of essential oil in relation to the initial raw material. For example, in the case of peppermint the concentration of essential oil in the granulate decreased by circa 71% in relation to the non-shredded material (1.08% and 0.31%, respectively).

In the case of analyses of dietary supplements containing peppermint leaf in their composition it was found that the average potential availability of essential oils supplied to the organism with products of the type of dietary supplements, for the recommended doses, was significantly lower than in the corresponding peppermint tea brews, as much as ca. 6-fold lower—the maximum value of potential availability of essential oil for peppermint tea is 96.60 mg (min. 5.62 mg), while the corresponding value for the dietary supplement is at the level of 23.12 mg (min. 0.08 mg).

Potential availability of essential oil for the analysed forms of dietary supplements was generally significantly different. Taking into account a minimum daily intake of essential oil, the highest availability of this substance can be noticed in tablets which contain peppermint leaves–9.40 mg (sMT1, sMT4, sMT9). However, this type of supplements contained the lowest amount of essential oil: 0.08 mg (sMT6). For the maximum daily intake, the highest potential availability of essential oil was present in the capsules–23.12 mg (sMT2) as well as the tablets and the liquid–18.80 mg (sMT1, sMT4, and sMT9).

Góra and Lis [13] report that approximately 150 components of peppermint essential oil have been identified to date, the main ones being menthol (20–80%) and menthone (15–45%).

The following components have been identified in the *M*. *piperita* essential oil: menthol (33–60%), menthone (15–32%), isomenthone (2–8%), 1,8-cineole (5–13%), menthyl acetate (2–11%), menthofuran (1–10%), limonene (1–7%), -myrcene (0.1–1.7%), -caryophyllene (2–4%), pulegone (0.5–1.6%) and carvone (1%) [14]. Taking into account the classification of those authors, the peppermint teas in the experiment belong to a chemotype rich in menthol, menthone and iso-menthone.

According to PP VIII [4], the levels of the main components of peppermint oil should fall within the following ranges: menthone 14–32%, iso-menthone 1.5–10%, menthyl acetate 2.8–10%, and menthol 30–55%.

The unprocessed raw material was characterised by the presence of the following main components isolated from the essential oil: menthol (23.4–29.5%), menthone (26.9–38.4%), menthyl acetate (7.8–15.8%) and iso-menthone (7.0–12.3%)–Table 4. The peppermint oils analysed differed very slightly in their chemical composition. The dominant components in oils from the peppermint teas included menthol (23.6–40.8%), menthone (26.0–41.7%), menthyl acetate (5.3–7.9%), iso-menthone (3.9–7.2%) and neo-menthol (4.1–7.4%).

**Table 4:** The main components of essential oils obtained from unprocessed materials and peppermint teas.

		cMT1^#^		cMT2		cMT3		cMT4		tMT1		tMT2		tMT3		tMT4		tMT5		tMT6		tMT7		tMT8	
Compound	IR	%	±SD	%	±SD	%	±SD	%	±SD	%	±SD	%	±SD	%	±SD	%	±SD	%	±SD	%	±SD	%	±SD	%	±SD
**1,8-cineole**	1037	2.6	0.1	4.5	0.0	2.5	0.2	3.2	0.1	2.6	0.1	2.1	0.1	1.7	0.0	2.4	0.1	1.4	0.0	1.6	0.0	2.0	0.0	1.8	0.0
**menthone**	1168	26.9	0.2	33.8	0.9	38.4	0.1	37.9	0.3	26.0	0.6	33.1	0.3	39.2	0.6	41.7	0.4	33.4	0.3	38.2	0.5	32.9	0.2	28.9	0.3
**iso-menthone**	1175	12.3	0.2	7.6	0.0	7.0	0.2	7.5	0.1	3.9	0.2	5.5	0.1	7.2	0.2	6.8	0.1	6.1	0.1	4.9	0.1	5.1	0.1	4.8	0.1
**neo-menthol**	1179	2.9	0.1	2.4	0.1	2.4	0.1	2.7	0.1	4.1	0.2	5.9	0.2	7.4	0.2	7.2	0.1	6.5	0.1	5.3	0.1	5.5	0.1	5.2	0.1
**menthol**	1193	29.5	0.3	28.0	0.5	26.9	0.2	23.4	0.2	39.4	0.5	36.2	0.1	25.8	0.4	23.6	0.5	33.1	0.1	32.1	0.2	37.1	0.6	40.8	0.5
**pulegone**	1249	1.4	0.0	0.1	0.0	-	-	0.3	0.0	1.9	0.1	0.5	0.0	0.6	0.0	0.5	0.0	0.6	0.0	0.8	0.0	0.7	0.0	1.0	0.0
**piperitone**	1266	1.3	0.0	1.2	0.0	tr.	-	0.7	0.1	3.3	0.1	1.0	0.0	1.3	0.0	1.3	0.0	1.4	0.0	1.0	0.0	1.1	0.0	1.5	0.0
**menthyl acetate**	1296	7.8	0.1	9.3	0.1	15.8	0.6	10.5	0.2	5.3	0.1	7.9	0.2	6.5	0.1	5.7	0.4	7.2	0.4	6.0	0.4	6.8	0.4	tr.	—
**germacrene D**	1489	1.4	0.1	2.0	0.1	1.5	0.0	3.7	0.1	1.9	0.0	1.7	0.0	2.5	0.1	2.7	0.1	2.5	0.1	2.6	0.4	1.5	0.0	1.1	0.0

^#^Designation according to Table 1.

IR- retention indices (from temperature-programming, using definition of Van den Dool and Kratz [10].

tr.—less than 0.1%.

In the oils isolated in the experiment the content of menthone was higher than the range indicated in the PP VIII [4] while the levels of iso-menthone and ethyl acetate were conforming to the PP VIII [4], and two oils were characterised by menthol levels below 30%.

Peppermint is used in EU countries both as food as a medicinal product. The use of the raw material in amounts commonly occurring in food products is safe. In the USA, peppermint has the status of GRAS (Generally Recognized as Safe, FDA). The material was commonly used as a food component in Poland before 1997. Extracts from leaves are used as medical potions for improving digestion, and with diastolic and cholagogic effects. Leaves of peppermint are a component of many herbal blends [15]. Peppermint is used for the production of peppermint oil whose main component is menthol [16]. A single medicinal dose is 3–6 g of raw material or 5–15 g of tincture [17], 1,5–3 g of raw material 3 times a day [16], 4–9 g of raw material daily (divided into portions) [18].

### Lemon balm

Essential oil yield in raw material—leaf of lemon balm varied from 0.17% to 0.19% (mean of 0.18%)–Table 2, while the level on essential oil in lemon balm teas varied within the range of 0.07–0.17% (mean of 0.12%). The pharmacopeial raw materials are dried leaves of *Melissa officinalis* L. The standard acc. to PP VI [5] states that leaves of lemon balm should contain not less than 0.05% of oil, and thus all of the herbal teas under analysis complied with that requirement, which indicates good quality of the used raw material. The lemon balm teas were characterised by, on average, one a half lower yield of essential oil compared to the unprocessed raw material. The mean potential availability of essential oil in dietary supplements with lemon balm, for the recommended doses, was four times lower than in the teas—the maximum value of potential availability of essential oil for lemon balm tea is 10.20 mg (min. 1.40 mg), while the corresponding value for the dietary supplement is at the level of 4.50 mg (min 0.04 mg).

For the minimum recommended daily intake of supplements containing lemon balm the statistically highest potential availability of essential oil can be found in the capsules–3.60 mg (sML10), and in powders–1.66 mg and 1.50 mg (sML5, sML9). The potentially lowest available amount of essential oil for minimum daily intakes was found in the capsules–0.04 mg (sML8), and the tablets–0.06 mg (sML1 and sML7). For the maximum daily intake, the highest potential availability of essential oil can be observed in the powder–4.50 mg (sML9)–3.78 mg and 3.60 mg (sML6 and sML10).

Góra and Lis [13] report that the qualitative composition of lemon balm oil is subject to notable variation and large differences were observed in the levels of the main components, these findings are supported by this study. The authors cited state that the dominant components of lemon balm oil are citronellal (11.31%), neral (22.18%) and geranial (33.6%). In lemon balm oils obtained from leaves and green parts of plants grown in Poland the following main components were assayed: β-caryophyllene (5.88–31.73%), caryophyllene oxide (0.01–12.2%), geranial (6.27–32.92%), neral (4.59–17.37%) and citronellal (1.33–15.18%) [19]. In essential oils isolated from lemon balm grown in Greece 45 components were identified, the dominant ones being beta-pinene, sabinene, E-caryophyllene and caryophyllene oxide [20].

The unprocessed raw material was characterised by the presence of the following main components isolated from the essential oil: geranial (8.6–32.1%), neral (4.1–20.6%), caryophyllene oxide (20.2–36.0%), E-caryophyllene (1.7–9.1%), citronellal (5.2–18.3%)–Table 5. In the analysed lemon balm teas 117 components of essential oil were identified. The qualitative composition of the lemon balm tea essential oils is quite similar, while quantitative variation was noted for the particular components. The dominant compounds in lemon balm oil from the teas are geranial (3.6–38.5%), neral (2.9–26.5%), menthyl acetate (1.0–37.4%), caryophyllene oxide (5.6–19.9%), E-caryophyllene (3.8–9.2%), citronellal (0.4–8.7%) (Table 5).

**Table 5:** The main components of essential oils obtained from unprocessed materials and lemon balm teas.

		cML1*		cML2		cML3		cML4		tML1		tML2		tML3		tML4		tML5		tML6		tML7		tML8	
Compound	IR	%	±SD	%	±SD	%	±SD	%	±SD	%	±SD	%	±SD	%	±SD	%	±SD	%	±SD	%	±SD	%	±SD	%	±SD
**limonene**	1033	-		0.1	0.0	-	-	-	-	1.4	0.0	1.0	0.1	0.4	0.0	1.8	0.2	0.8	0.1	4.4	0.2	1.0	0.1	1.9	0.1
**citronellal**	1158	-		8.5	0.2	18.3	0.1	5.2	0.2	0.4	0.0	2.1	0.1	2.3	0.1	6.8	0.3	4.2	0.1	8.7	0.2	2.1	0.0	8.4	0.1
**menthone**	1164	5.6	0.2	2.9	0.0	3.5	0.3	-	-	6.4	0.1	2.5	0.1	1.4	0.0	5.6	0.2	1.5	0.0	4.2	0.2	2.5	0.0	3.4	0.1
**iso-menthone**	1173	0.9	0.0	0.7	0.1	2.3	0.2	-	-	1.8	0.1	1.0	0.1	0.5	0.0	3.0	0.1	0.8	0.0	1.3	0.1	1.0	0.0	1.4	0.0
**iso-menthol**	1186	11.4	0.4	1.5	0.1	1.9	0.2	-	-	4.9	0.2	3.8	0.1	1.1	0.1	6.0	0.3	1.6	0.2	5.8	0.3	3.8	0.1	3.1	0.1
**neral**	1247	4.1	0.8	10.0	0.5	14.8	0.7	20.6	0.2	4.1	0.1	2.9	0.0	26.5	0.4	4.8	0.1	14.0	0.3	5.7	0.1	2.9	0.1	11.0	0.2
**geraniol**	1255	-	-	-	-	-	-	0.1	0.0	4.1	0.1	0.4	0.0	-	-	-	-	-	-	tr.	—	0.4	0.0	-	-
**geranial**	1276	8.6	0.5	14.5	0.6	18.2	0.9	32.1	0.4	6.0	0.0	3.6	0.2	38.5	0.6	6.0	0.6	23.0	0.3	7.6	0.2	3.6	0.1	16.6	0.4
**isobornyl acetate**	1296	-	-	-	-	-	-	-	-	5.7	0.2	2.6	0.0	0.4	0.0	-	-	-	-	2.0	0.1	2.6	0.2	tr.	—
**menthyl acetat**	1303	-	-	-	-	-	-	-	-	1.4	0.0	34.2	1.0	1.0	0.0	6.5	0.2	7.7	0.4	12.6	0.4	37.4	1.1	2.7	0.3
**carvacrol**	1310	-	-	-	-	-	-	-	-	0.3	0.0	2.00	0.0	0.2	0.0	1.8	0.0	3.7	0.3	1.8	0.3	2.0	0.0	1.1	0.1
**eugenol**	1364	-	-	-	-	-	-	-		3.9	0.2	tr.	-	tr.	-	tr.	-	tr.	-	tr.	-	tr.	-	tr.	-
**E-caryophyllene**	1427	7.8	0.9	9.1	0.2	7.9	0.1	1.7	0.0	7.8	0.3	3.8	0.1	4.0	0.2	5.1	0.2	9.2	0.5	4.1	0.3	3.8	0.1	8.2	0.3
**caryophyllene oxide**	1594	36.0	0.1	20.2	0.2	21.4	0.2	21.6	0.8	19.9	0.3	6.0	0.1	5.8	0.2	14.1	0.5	7.0	0.1	5.6	0.6	6.0	0.1	9.2	0.6
**humulene epoxide II**	1620	2.6	0.3	1.3	0.0	1.0	0.0	1.7	0.1	-	-	-	-	-	-	-	-	-	-	-	-	-	-	-	-
**14-hydroxy-9-epi-(E)-caryophyllene**	1685	2.7	0.4	4.5	0.1	1.1	0.0	0.4	0.0	-	-	-	-	-	-	-	-	-	-	-	-	-	-	-	-

^#^Designation according to Table 1.

IR- retention indices (from temperature-programming, using definition of Van den Dool and Kratz [10].

tr.—less than 0.1%.

Lemon balm has sedative, carminative, diaphoretic and antiviral properties. These substances are utilized to help with cases of insomnia that are related to nervous disorders as well as to the functional disorders of the stomach and intestines [15]. A single therapeutic dose is 1.5–4.5 g of raw material for a cup of infusion, taken several times a day as required [21, 22], 2–3 g: taken two to three times a day [23]. Lemon balm is used in the EU countries both as a food and a medicinal product. The use of the raw material in amounts commonly occurring in food is safe. In the USA, lemon balm has gained the status of GRAS (Generally Recognized as Safe, FDA). The raw material was commonly used in Poland as a food component before 1997.

### Chamomile

The yield of essential oil in unprocessed raw material—inflorescence of chamomile was from 0.41% to 0.74% (mean of 0.51%)–Table 2. The concentration of the oil in the chamomile teas being investigated here varied within the range from 0.33 to 0.59% (mean of 0.48%) (Table 2). The pharmacopoeial raw material consists of the dried anthodia of chamomile *Matricaria recutita* L, standardised according to the PP VIII [4] for essential oil yield at the level of 0.4%. Chamomile anthodia contain up to 1.5% of oil [24]. Six out of the eight analysed chamomile teas met the requirements of PP VIII [4].

Taking into account the minimum recommended daily intake of supplements containing chamomile the statistically highest availability of essential oil was found in the powder–7.20 mg (sMC9), and in the capsules–6.24 mg (sMC10). The significantly lowest potential amount of the essential oil for minimum daily intakes were found in the capsules–0.67 mg and 0.71 mg (sMC3, sMC2), and in the tablets–0.72 mg (sMC6). Taking into account the maximum daily intake, the highest potential availability was evidenced by the powder–21.60 mg (sMC9).

An earlier study concerned with the processing of chamomile raw material into granulate revealed significant losses of essential oil when compared to the initial raw material, reaching the level of ca. 44% [12]. It was found that the average potential availability of essential oils in products of the type of dietary supplements, for the recommended doses, is circa three times lower than in the equivalent herbal teas. The smaller differences in essential oil content yield in chamomile tea relative to the initial raw material may be related to the kind of secretory structures that in case of chamomile take the form of internal structures, as opposed to the secretory structures of peppermint and lemon balm which have the character of external secretory trichomes and are thus more sensitive to various destructive effects inherent to food processing.

According to the scientific literature available on the topic, the dominant components of chamomile oils are α-bisabolol (1–60%), α-bisabolol oxide A (2%-60%), α-bisabolol oxide B (3–50%), chamazulene (2–25%), α-bisabolone oxide A (0.4–12%) and E-β-farnesene (5–40%), those values are duly confirmed by the results of the study presented here [12, 13, 25, 26]. Góra and Lis [13] classify several chemotypes of chamomile.

The dominant components in essential oil isolated from unprocessed inflorescence of chamomile are α-bisabolol oxide A (21.7–41.8%), α-bisabolol oxide B (16.7–30.0%), α-bisabolon oxide A (9.9–19.6%), chamazulene (9.6%-18.3%)–Table 6. In the chamomile teas 95 compounds included in the composition of essential oils were identified. In terms of the qualitative composition of essential oil the particular teas did not differ much from each other. The main components of the oils identified are α-bisabolol oxide A (17.4–47.2%), α-bisabolol oxide B (17.8–37.5%), α-bisabolone oxide A (0.3–16.9%), chamazulene (2.7%-13.9%), E-β-farnesene (1.0%-11.4%) (Table 6).

**Table 6:** The main components of essential oils obtained from unprocessed materials and chamomile teas.

		cMC1^#^		cMC2		cMC3		cMC4		tMC1		tMC2		tMC3		tMC4		tMC5		tMC6		tMC7		tMC8	
Compound	IR	%	±SD	%	±SD	%	±SD	%	±SD	%	±SD	%	±SD	%	±SD	%	±SD	%	±SD	%	±SD	%	±SD	%	±SD
**E-beta-farnesene**	1457	3.0	0.1	1.5	0.1	0.1	0.0	2.3	0.1	1.0	0.1	4.7	0.2	8.3	0.4	8.4	0.8	7.8	0.5	11.4	0.7	9.1	0.6	3.7	0.2
**spathulenol**	1589	5.4	0.2	4.6	0.0	2.9	0.1	3.2	0.1	1.2	0.1	2.9	0.0	2.0	0.0	5.2	0.3	7.0	0.3	0.3	0.0	2.1	0.0	2.9	0.0
**α-bisabolol oxide B**	1667	30.0	0.2	18.4	0.1	26.9	0.2	16.7	0.2	33.6	0.2	34.9	0.1	17.8	0.7	26.6	0.8	31.4	1.2	29.1	0.5	21.8	0.1	37.5	0.8
**α-bisabolone oxide A**	1695	19.6	0.2	10.6	0.1	10.2	0.1	9.9	0.1	16.9	0.4	15.8	0.3	11.8	0.5	12.7	0.9	15.4	0.1	13.5	0.5	11.1	0.6	0.3	0.0
**chamazulene**	1748	9.6	0.2	13.9	0.4	18.3	0.6	17.4	0.1	13.9	0.2	3.5	0.1	2.7	0.1	3.1	0.2	8.8	0.1	4.1	0.1	7.5	0.2	13.1	0.3
**α-bisabolol oxide A**	1765	21.7	0.3	41.0	0.1	30.8	0.8	41.8	0.3	27.0	0.2	28.7	0.4	47.2	1.1	31.9	0.3	17.4	0.3	22.5	0.5	24.5	0.5	27.5	0.5

^#^Designation according to Table 1.

IR- retention indices (from temperature-programming, using definition of Van den Dool and Kratz [10]

tr.—less than 0.1%.

In chemotype A the dominant compound is α-bisabolol oxide A (Egyptian, Slovak, Hungarian and other European chamomiles) [13], and three of the chamomile teas tested (tMC3, tMC4, tMC7) are classified as such. Chemotype B is characterised by a dominance of α- bisabolol oxide B (Argentine chamomile oil) [13], the remaining five teas analysed being classified in that chemotype. Therefore, it can be assumed that the raw materials used for their production had been imported.

Chamomile and its extracts display diastolic and carminative effects. Water extracts have an immuno-stimulating effect. It is used to treat digestive disorders, inflammatory conditions of the gastrointestinal duct and of the enterospasms. Infusions from chamomile anthodia normalise the work of the intestines and facilitate their peristalsis [15]. In gastrointestinal disorders it is recommended to use, 3–4 times a day, a cup of infusion prepared from 3 g of chamomile flowers drowned in hot water [27, 28]. Chamomile inflorescence is used in the EU countries both as a food and a medicinal product. The raw material was commonly used in Poland as a food component before 1997.

When considering the overall results that have been obtained it should be emphasised that peppermint, lemon balm and chamomile teas are a more attractive source of essential oils for our organism than dietary supplements. This fact stems from the lower content of the herbal raw material present in dietary supplements, whose content is many times lower than the therapeutic dose. Moreover, the herbal component of a dietary supplement is subjected to a variety of technological operations (production of tablets, capsules, liquids, powders) which cause a reduction in the overall concentration of essential oil. Significant differences in the levels of the potentially available essential oils in recommended daily dietary supplement intakes are due to the fact that there are no guidelines regulating the content of health- promoting raw material in dietary supplements, with the tendency of having a lower level than the one present in the corresponding medications, which enables the manufacturers to use a big amount margin as far as the relevant ingredients are concerned. The research does not show clear trends regarding the highest amount of potentially available essential oil in a given dietary supplement. Among analysed forms such as tablets, capsules, powders and liquids, when taking into account the potential availability of essential oils, the ones showing the highest values are the powders, which results from the bigger amount of plant raw material being included in a recommended daily intake. However, powders are not appealing to customers, which is visible in the analysed range of supplements, where only 3 out of 30 products were sold in the powder form, whereas the rest was in the form of tablets–11, capsules–10 and lotions –6.

The various forms of dietary supplements such as tablets, capsules, extracts, are all very convenient for the consumer and at the same time they resemble medical potions, which may increase their perceived effectiveness in contrast to food products in the form of traditionally brewed tea bags [29]. In spite of the detailed information provided on the label or the attached description concerning the content of the herbal component, the consumers may still have problems with the correct interpretation of the potential availability of a biologically active component when confronted by other products such as herbal teas [30–32]. Dietary supplements, in spite of their significantly higher prices compared to such food products as herbal teas, enjoy a high demand among the consumers and the market for such products is constantly growing [33]. However, it is important to raise the awareness of the consumers so that they can make conscious and informed choices taking into account not only the attributes related to the simple usage of the products, but also considering the notably more important factor which undoubtedly is the potential availability of active substances present in a given product.

## Conclusions

1. Analysis of the composition of the dietary supplements showed that they contain on average significantly lower amounts of plant material compared to the herbal teas.
2. It has been shown that the tested herbal teas are characterized by a different yield of the essential oils with an average that is lower than is present in the raw feedstock.
3. It was found that the average potential availability of essential oils in the products such as dietary supplements for the doses recommended by the producer is lower than in the corresponding tea infusions.
4. The essential oils extracted from herbal teas have similar chemical profiles with the individual components of the quantitative variation characteristics for the raw materials.
